# The impact of the microbiome in cancer: Targeting metabolism of cancer cells and host

**DOI:** 10.3389/fonc.2022.1029033

**Published:** 2022-11-16

**Authors:** Jia-Ting Huang, Yu-Qin Mao

**Affiliations:** ^1^ Center for Traditional Chinese Medicine and Gut Microbiota, Minhang Hospital, Fudan University, Shanghai, China; ^2^ Institute of Fudan-Minhang Academic Health System, Minhang Hospital, Fudan University, Shanghai, China

**Keywords:** tumor, microbiome, metabolism, host, therapy

## Abstract

Abnormal metabolic alterations of cancer cells and the host play critical roles in the occurrence and development of tumors. Targeting cancer cells and host metabolism can provide novel diagnosis indicators and intervention targets for tumors. In recent years, it has been found that gut microbiota is involved in the metabolism of the host and cancer cells. Increasingly, gut microbiome and their metabolites have been demonstrated great influence on the tumor formation, prognosis and treatment. Specific gut microbial composition and metabolites are associated with the status of tumor in the host. Interventions on the gut microbiota can exert the protective effects on the tumor, through the manipulation of structure and its related metabolites. This may be the new approach to improve the efficacy of tumor prevention and treatment. Here, we discuss the effects and the underlying mechanisms of gut microbiota and microbial-derived metabolites in tumor progression and treatment.

## 1 Altered metabolism and cancer

One of the hallmarks of cancer is altered metabolism (1). Cancer-associated mutations change the signaling pathways of regulating cell metabolism, resulting in rewiring of cancer metabolism and reshaping of cancer cells, favoring the growth of rapidly proliferating cancer cells (2). Mounting studies have demonstrated that changes in metabolic signaling pathways can regulate tumor signal transduction pathways, thereby affecting the occurrence and development of tumors (3).

Available evidence from epidemiologic investigations, preclinical experiments, and clinical studies demonstrate that the metabolic syndrome, including obesity, hypertension, dysglycemia have become an important etiologic factor for the development and progression of common cancer (4, 5). Studies have shown that an increase in waist circumference or body mass index (BMI) significantly increases the risk of various types of cancer, including breast cancer, ovarian cancer, liver cancer (6, 7). Evidences show that obese patients with cancer have unfavorable prognosis and outcome, resulting from poorer response to treatment (8).

Metabolic disorders can result in rewiring of cancer cells metabolism, and metabolites can reshape the microenvironment through different metabolic signaling pathways, thereby synergistically promoting the occurrence and development of tumors (9). The development and progression of tumors can lead to metabolic reprogramming, but metabolic disorders precede the development of tumors. Therefore, metabolic abnormalities are one of the key factors in tumorigenesis. Therefore, cancer can be regarded as a metabolic disease, to some extents. It deserves further studies to find out targets in metabolism that affect tumor development. Targeting metabolic pathways is considered as a promising approach for cancer therapy.

Recently, studies have demonstrated that gut microbiota is composed of a complex microecosystem that contributes to the health of the host. The balance of metabolism in host is related to the composition and function of gut microbiota. Gut microbiota directly involves in the metabolism and the evolution of the host (10). The interaction on the host and gut microbiota is closely related to metabolism and immunomodulatory functions (11). This review will summarize the targets of gut microbiota to regulate metabolism that affect the occurrence and development of tumor.

## 2 Gut microbiota and human health

### 2.1 Gut microbiota

The host is home to an ecological community of trillions of symbiotic microorganisms that are essential for maintaining the homeostasis and health in the host. In 2001, Joshua Lederberg named the consortium of microbes as the “Microbiome” (12). The commensal bacteria or the component of the “human flora” is the dynamic residents. The development of commensal microbiomes (such as bacteria, archaea, fungi, and viruses) is influenced by external and internal factors occurred in the host throughout the early life (13). So far, the gut microbiome is one of the most popular and emerging systems that appeal to researchers, especially its role in regulating the host’s health.

The human gut contains more than 3x10 (13) bacteria, including a repertoire of five phyla with *Firmicutes*, *Bacteroidetes*, *Actinobacteria*, *Proteobacteria* and *Verrucomicrobia*. The gut microbiome is 150 times larger than the entire human genome, considered as the “second genome” of human, as its vital role in the health of host. The composition of the microbiota is determined and influenced by many factors such as host genetics, different lifestyle, incidence of diseases, and exposure to antibiotics (14). The structure of host microbiome has been developing during the initial years of life. Since then, the composition of the gut microbiota and other epithelial barriers remain relatively stable throughout the adulthood. However, diet, changes in lifestyle, diseases and associated treatments can affect the composition of gut microbiota. The microbiota locates in the epithelial barrier of the gut, which can influence the local and systemic metabolic functions, inflammation, and immunity. Therefore, it involves in regulating the initiation and progression of tumor, and the response to anticancer therapies (15).

Emerging research highlights the diverse functions of the gut microbiota in the health of host. Gut microbiota regulates many essential biological processes, such as monitoring epithelial development, metabolic functions, and innate immune responses including activation and maturation of immune cells, prevention of systemic migration and infiltration of enteric pathogens (16). However, disrupting the existing gut microbiota is referred as microbial dysbiosis. The gut microbial imbalance is strongly associated with many metabolic diseases such as cardiovascular diseases, diabetes, obesity, and even a variety types of cancer (17). It is reported that commensal bacteria, such as *Clostridium difficile* and vancomycin-resistant *Enterococcus*, acquire pathogenicity by interrupting the ecology of intestine (18). This suggests that the imbalance of gut microbiota can lead to the occurrence of diseases.

Interestingly, the pathogenic microbiota in the gut of host often cause more than 20% of cancers, such as pathogenic *Escherichia coli* (*E. coli*) which contributes to colorectal cancer (CRC) (19). It deserves further studies to demonstrate the association and functional impact of gut microbiota on the health of host. Significant alterations of microbial community have been revealed by metagenomic sequencing in patients living with cancer, compared with those in control. Moreover, intestinal microbes colonize in tumors and play an important role in the pathogenesis, especially by inducing the synthesis of nutrients in the host through their metabolites, and regulating the efficacy of anti-tumor therapy and the clinical outcome (20). Furthermore, commensal flora can regulate the occurrence and development of tumors through modifying the immune system of host, as the immune deficiency is one of the important markers of cancer pathogenesis (21).

### 2.2 Gut microbiota and tumor

**Figure 1:**
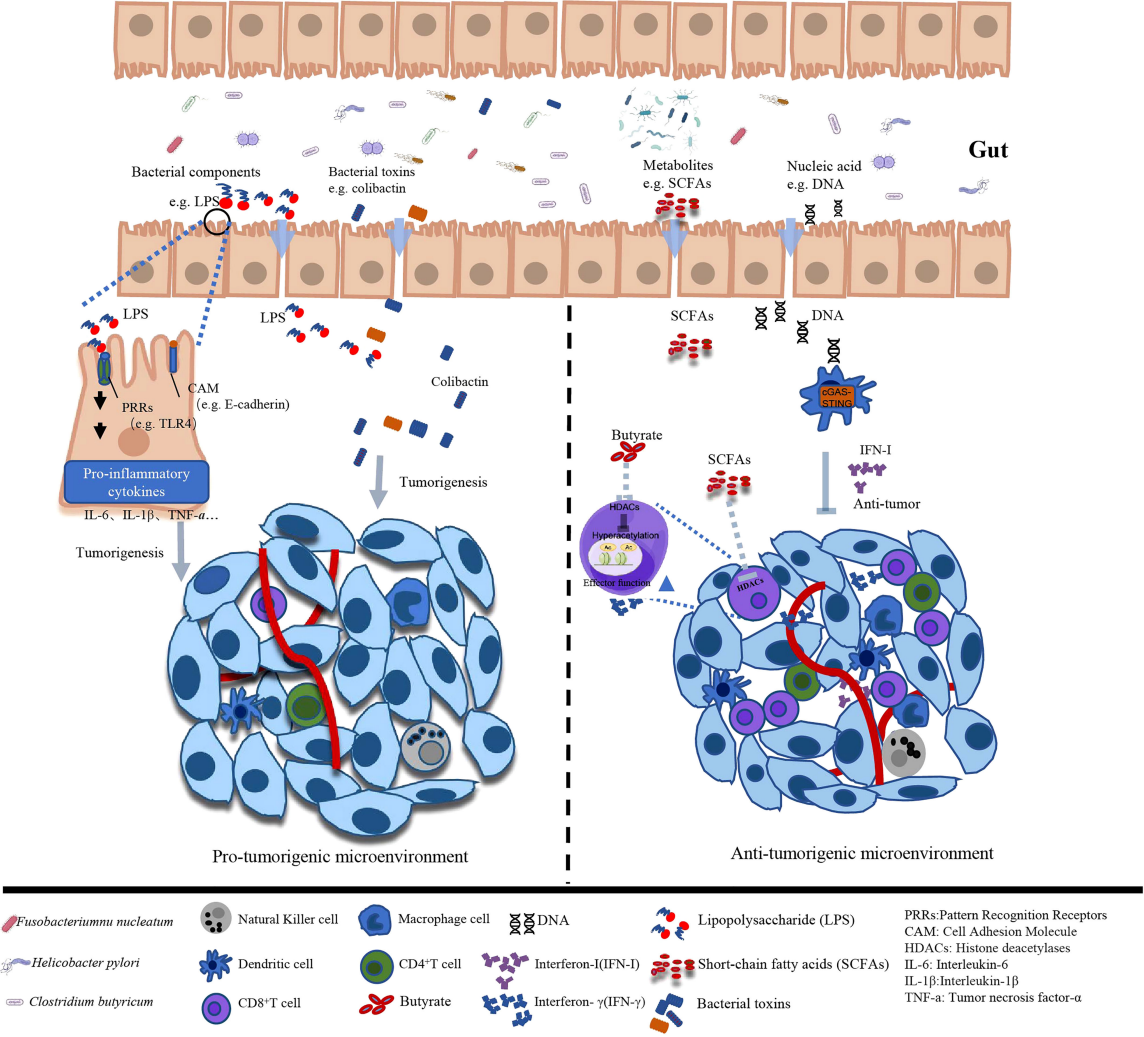
Different effects of gut microbiota and associated metabolites on tumor microenvironment.

The interactions between microbes and host cells are essential for the local and systemic health and the regulation of many physiological functions (20). Importantly, the gut microbiota can also systematically influence the metabolism, immunity and homeostasis of host to affect cancer development and treatment response (22) (**Figure 1**). The subtle and well-managed balance of system functions result from the delicate interactions between the host and gut symbionts, such as energy balance, metabolism, inflammation and immunity. Various members of gut microbiota, including *Bacteroides*, *Lactobacillus*, and *Bifidobacterium*, are closely associated with the metabolism of dietary fiber that prove difficult to catabolize in the gastrointestinal tract of the host.

Many studies have shown that many diseases, including cancer, may be induced by the dysbiosis of the microbiota. Importantly, microbial pathogens are responsible for high morbidity of cancers (23). Therefore, it is of great significance to investigate the role of these microbial pathogens in tumorigenesis and development. An imbalanced gut microbiota is associated with numerous host pathologies, and a well-balanced composition of gut microbiota is essential for the host’s health. Thus, disturbances in the gut microbiome can exacerbate the development of cancers (**Table 1**).

**Table 1:** The effect of substances of gut microbiota in cancer.

Study	Microorganism	Effector	Effect	Cancer type
Singh, A. et al.	*Propionibacterium acnes* (*P. acnes*) (24)	Secreting proinflammatory cytokines such as IL-6, IL-8 and TNF-α	Pro-cancer	Prostate Tumor
Pere-Vedrenne, C. et al.	*Helicobacter hepatica* (*H. hepaticus*) (25)	cytolethal distending toxin (CDT)	Pro-cancer	Hepatocellular cancer
Moss, S. F. et al	*Helicobacter pylori* (*H. pylori*) (26)	Vacuolar toxin A (vacA) and cytotoxin-related gene A (CagA)	Pro-cancer	Gastric cancer
Williamson, A. J. et al.	*Enterococcus faecalis* (*E. faecalis*) (27)	GelE	Pro-cancer	Colorectal cancer
Wilson, M. R. et al	*Escherichia coli* (*E. coli*) (28)	Colibactin	Pro-cancer	Colorectal cancer
Mima, K. et al.	*Fusobacterium nucleatum* (*F. nucleatum*) (29)	FadA; Fap2 and LPS	Pro-cancer	Colorectal cancer

FadA, for Fusobacterium adhesin A; IL-6, interleukin-6 (IL-6); IL-8, interleukin-8 (IL-8); TNF-α, tumor necrosis factor-α.

LPS, Lipopolysaccharide.

Studies have found significant differences in the composition of gut microbiota between patients with CRC and healthy controls. The structure of gut microbiota in patients with CRC is in dysbiosis, in which the relative abundance of *Fusobacterium* sp., *Pavimonas micra*, *Peptostreptococcus stomatis* were significantly over-represented (30, 31). A meta-analysis of 526 fecal metagenomic data reveals seven major clusters of enriched bacteria in patients with CRC, namely, *Bacteroides fragilis, Fusobacterium nucleatum, Porphyromonas asaccharolytica, Parvimonas micra, Prevotella intermedia, Alistipes finegoldii, and Thermanaerovibrio acidaminovorans* (32). Studies demonstrate that *E. coli*, *Bacteroides fragilis*, and *Peptostreptococcus anaerobius* have oncogenic properties through inducing genotoxic stress, cholesterol biosynthesis, and activating Th1 immune responses. However, the abundance of butyric-producing bacteria *Clostridium butyricum* and lactic-acid-producing bacteria *Streptococcus thermophiles* are down-represented (33). Preclinical studies have also shown that the gut microbiota plays a crucial role in the development of CRC. The known carcinogen, Cycads, failed to induce tumors in the germ-free rats. In addition, when 1, 2-dimethylhydrazine was administered to rats, 93% of normal rats developed tumors, while only 21% of germ-free rats developed cancers (34). Subsequent studies have shown that the gut microbiota, including specific *E. coli* strains, *Enterococci*, *Bacteroides*, and *Clostridium genera*, promote the development of cancer. In addition, transplantation of fecal stools from patients with CRC into germ-free mice can promote tumor formation (35). There are 21 bacterial strains enriching significantly in patients with gastric carcinoma, including *Fusobacterium nucleatum* (*F. nucleatum*), *Parvimonas micra*, *Streptococcus anginosus* and *Peptostreptococcus stomatis*. In patients with pancreatic cancer (PC), the abundance of *Porphyromonas*, *Streptococcus*, *Bifidobacteria* and *Fusobacteria* were over-represented, which was demonstrated by 16S rRNA sequencing. A metagenomic study found that the presence of oral bacteria, *Haemophilus*, *Porphyromonas*, *Cilia*, and *Fusobacteria* is correlated with the increased risk of PC (36). These studies identify the vital role of gut microbiota to induce cancer.

*Propionibacterium acnes* (*P. acnes*), is the most abundant microorganism in prostate tissues. Cavarretta et al. reveal that *P. acnes* is over-represented in prostatic tumor tissues, compared to non-tumor tissues (37). Cohen et al. demonstrate a significantly higher degree of inflammation in *P. acnes*-positive prostate tissues (38). *P. acnes* promotes the proliferation of prostate epithelial cells by secreting proinflammatory cytokines such as interleukin-6 (IL-6), interleukin-8 (IL-8) and tumor necrosis factor-α (TNF-α) (24). Therefore, studies have revealed that *P. acnes* infection may be a cause of a long-term of pathogenic cascade, an inflammatory process in the prostate tissues.

*Helicobacter hepatica* (*H. hepaticus*) is the specie isolated from the liver and intestine of inbred mice (39). Accumulating evidence indicates *H. hepaticus* and related species play important roles in promoting intestinal dysbiosis. *H. hepaticus*-induced intestinal dysregulation can promote the development of breast and prostate cancers, possibly by modulating the innate immunity (40). Ge et al. have revealed that infection of *H. hepaticus* may trigger precancerous lesion of CRC (41). Powrie et al. demonstrate that *H. hepaticus* produces a large soluble polysaccharide that can induce IL-10 production by intestinal macrophages that activates a specific anti-inflammatory signaling pathways (42). It is reported that the colonization of *H. hepaticus* contributes to the promotion of aflatoxin- and hepatitis C virus (HCV) transgene-induced hepatocellular carcinoma (43). In addition, the cytolethal distending toxin (CDT), as the main virulence factor of *H. hepaticus*, results in DNA double-strand breaks, may contribute to the expression of oncoprotein (25). As the pathogenic bacterium, *H. hepaticus* is a strong risk factor associated with hepatocellular carcinoma, prostate cancer, and breast cancer (44).

*Helicobacter pylori* (*H. pylori*), as a cancer-promoting microorganism, can lead to the formation of gastric cancer (26). Its main functional mechanism includes secretion of virulence factors, vacuolar toxin A (vacA) and cytotoxin-related gene A (CagA), and activation of multiple pathways, including epidermal growth factor receptor (EGFR) pathway, S6 kinase pathway and cell cycle progression pathway, to abnormally transform cell proliferation, cell cycle transition and cell death, thereby promoting cancer development (45). Other indirect mechanisms are reported as well. For example, *H. pylori* and *Enterococcus faecalis* (*E. faecalis*) can induce the generation of oxidative stress, leading to carcinogenesis. This helps promote sudden genomic instability and manipulate host immune responses, thereby tumor cells can evade immune surveillance. *E. coli* produce toxins, such as Colibactin, exhibit DNase activity causing DNA damage, ultimately leading to abnormal cell cycle and tumor progression (28).

*F. nucleatum*, a kind of oral bacteria, can promote cancer growth by activating Wnt signaling pathway (46). Actually, the virulence factor FadA (for *Fusobacterium* adhesin A) of *F. nucleatum* increases the expression of annexin A1 through E-cadherin signaling. Importantly, FadA upregulates c-Myc and cyclin D1 through activating the Wnt/β-catenin signaling pathway. In addition, other virulence factors, Fap2 and Lipopolysaccharide (LPS) from *F. nucleatum*, can also promote the transformation of normal epithelial cells into tumor cells (29). In addition, *F. nucleatum* attenuates T cell-mediated immune responses in CRC. The outer membrane protein Fap2 can also activate Toll-like receptor 4 (TLR4), leading to activation of nuclear factor-κB (NF-κB) and subsequent expression of oncogenic microRNA21 (47). Studies have revealed that supplementation with *F. nucleatum* for Apc^Min/+^ mice have smaller intestinal and colorectal tumors, compared with controls. Importantly, the activated NF-κB pathway leads to the over-expression of multiple proinflammatory cytokines, such as TNF, IL-6, IL-8, and interleukin-1β (IL-1β). Recent studies have shown that *F. nucleatum* significantly upregulates the expression of lncRNA keratin 7-antisense (KRT7-AS) and keratin 7 (KRT7) in CRC, thereby promoting cell migration and metastasis (48). Therefore, the abundance of *F.nucleatum* in feces may be a biomarker for noninvasive screening of CRC. In addition, the detection of serum IgA or IgG antibodies against *F. nucleatum* may provide the strategies of diagnosis. However, recent study has shown that *F.nucleatum* can enhance the efficacy of tumor immunotherapy (49).

In addition to its role in tumorigenesis, the gut microbiota has been implicated as the mediators in the response and outcome of tumor therapy. Studies have shown that regulating of gut microbiota can improve the efficacy of chemotherapy and reduce the side effects of chemotherapy.

## 3 Gut microbiota and cancer therapy

### 3.1 Gut microbiota and chemotherapy

Chemotherapy is one of the main therapies for various malignant tumors. These drugs can inhibit the growth and proliferation of tumor cells. Chemotherapy can kill tumor cells as well as normal tissue cells, which gives rise to some adverse effects such as nausea, vomiting, abdominal pain, diarrhea, intestinal mucosal ulcer bleeding.

Recent studies have shown that chemoradiotherapy can modulate the balance of intestinal microecology and induce intestinal damage. 16S rRNA sequence analysis demonstrates that the composition of gut microbiota is dysregulated in patients between patients before and after radiotherapy, of which the relative abundances of *Bacilli* and *Actinomycetes* are over-represented (50). Meanwhile, chemotherapy agents for cancer patients can directly destroy the structure of gut microbiota, leading to the imbalance of gut microbiota. Chemotherapy agents significantly reduce the abundance of *Lactobacillus* and *Bifidobacterium*, and increase the abundance of pathogenic bacteria, such as *E. coli*, which can lead to adverse effects of digestive tracts. Chemotherapy-induced pain occurs in 30% of cancer patients. However, the sensitivity of oxaliplatin-induced mechanical pain is found alleviated in germ-free and antibiotic-pretreated mice. And the fecal microbiota transplanted to germ-free mice lost its protective effect (50).

The gut microbiota modulates the responses to cancer therapy through a variety of mechanisms, including immune regulation, bacteria translocation, and enzymatic degradation (51). Translocation refers to the migration of commensal microbiota and pathogenic bacteria that crosses the intestinal epithelial barrier to induce systemic effects and regulates the efficacy of chemotherapy. *Paulos* et al. firstly report the beneficial role of the gut microbiota in cancer therapy, showing that whole-body radiotherapy induces translocation of gram-negative microbiota and promotion of LPS release, thereby inducing TLR4-dependent activation of antigen-presenting cells and enhancing the efficacy of adoptive T-cell therapy (52).

Cyclophosphamide (CTX) is widely used in tumor chemotherapy and exerts anti-tumor effects by interfering with the immune signaling cascades. CTX treatment is only effective in the presence of an intact gut microbiota. Preclinical studies have found that CTX-induced immune activation requires the participation of certain bacterial species, such as *Lactobacillus murinus* and *Lactobacillus johnsonii*. CTX treatment can lead to translocation of these bacteria to the lymph nodes and spleen, where can stimulates the host immune responses (53). Subsequent studies also reveal that *Enterococcus hirae* and *Barnesiella Intestinihominis* are required for the anti-tumor effects of CTX (54). In addition, antibiotic treatments result in a significantly reduction in the efficacy of chemotherapy, whereas germ-free animals do not respond to chemotherapy. Intestinal commensal bacteria, such as *Lactobacillus acidophilus*, play an important role in the anti-tumor effects of cisplatin and oxiplatin (55).

Clinical trials have shown that oral probiotic combinations (including *Lactobacillus plantarum*, *Bifidobacterium animalis*, *Lactobacillus rhamnosus*, and *Lactobacillus acidophilu*sto) can significantly alleviate oral mucositis caused by chemoradiotherapy in patients with nasopharyngeal carcinoma (56). Intestinal mucositis, caused by chemotherapy, can be alleviated by *Bifidobacterium infantis* supplementation in mice. Reasonable supplementation of probiotics and prebiotics, as well as changes of dietary habits can regulate gut microbiota, can improve the efficacy of chemotherapy, and reduce chemotherapy-induced side effects.

### 3.2 Gut microbiota and immunotherapy

Drug resistance, tumor recurrence and metastasis are the most common challenges in cancer therapy. Cancer can be also considered as the consequence of immune escape of the cancer cells (57). It is known that the immune system plays a dominant role in cancer defense, and its absence may greatly contribute to the onset and progression of cancer. Therefore, the main purpose of cancer immunotherapy is to improve immune response and overcome immune suppression. However, despite remarkable breakthroughs in recent years, the response to immunotherapy is heterogeneous and some immune-related adverse events have been identified. Therefore, the immunotherapy may not be effective and durable in all patients with cancer (58). Research have demonstrated the gut microbiome interacts strongly with the immune system, and the efficacy and toxicity of cancer immunotherapy are regulated by gut microbiome. The potential mechanisms underlying the unsatisfactory response to immune checkpoint inhibitors (ICIs) may result from the dysregulation of gut microbiota. However, the relationship between the gut microbiota and immunotherapy is under investigation.

Immune activation induced by CTLA-4 treatment depends on intestinal mucosal damage, microbiota dysregulation, and translocation of specific bacteria (*Bacteroides thetaiotaomicron* and *Bacteroides fragilis*), which can induce the activation of IL-12, proliferation of dendritic cells (DCs), and activation of specific Th1 cells. *Bifidobacterium* can activate CD11c^+^ DCs to enhance the efficacy of PD-L1 therapy. Several studies have demonstrated the vital role of gut microbiota in ICIs treatment for melanoma. Antibiotics treatment can destroy the efficacy of Immunolab, and the anti-tumor effect of the agents can be rescued by recolonizing germ-free or antibiotic-treated mice with the microorganism (59, 60). Patients with specific microbiota, such as *Bacteroides* and *Bifidobacteria*, have a better response to immunotherapy (61). Routy et al. analyze 249 patients, including types of non-small cell lung cancer (NSCLC), renal cell carcinoma, urothelial carcinoma, who have received second-line therapy with or without PD-1/PD-L1 inhibitors, and demonstrate that 69 patients who have recently taken antibiotics after receiving ICIs treatment, progression-free survival (PFS) and overall survival (OS) are significantly shortened. Furthermore, they analyze the gut microbiota in patients who was well-responded to ICIs treatment or not and the results reveal that *Akkermansia muciniphila* (*A. muciniphila*) was prevalent in patients who had achieved remission after ICIs treatment (62). Gopalakrishnan et al. involve 112 patients with advanced melanoma who have received anti-PD-1 immunotherapy into the study, analyze the diversity of gut microorganisms in patients, and reveal that the abundances of *Faecalibacterium* and *Clostridiales* are related to the efficacy of PD-1 therapy (61). *Matso*n et al. find that compared with 26 melanoma patients who do not respond to immunotherapy, 16 responders have higher abundance of *Bifidobacterium longum*, *Collinsella aerofaciens* and *Enterococcus faeciumre (* 59).

The gut immune system consists of immune cells and immune molecules in the epithelium and lamina propria, intestinal collecting lymph nodes (Peyer’s patches) and mesenteric associated lymph nodes. As a direct consequence of the mutualism between the gut microbiota and the host, gut microbiota can determine the immune system status of the gut, promote and regulate innate and adaptive immunity.

## 4 Manipulation of gut microbiome affects the occurrence and progression of tumor

Previous studies have demonstrated that regulating the composition of gut microbiota can affect the occurrence and progression of tumors, as well as the response to anti-tumor therapy. Specific gut microbiota can promote tumor formation, or affect the immunity of the host, and thus exert effects on tumor progression and treatment. Therefore, intervention targeted on gut microbiota is expected to achieve precise treatment on tumors. At present, the main intervention approaches for microbiota are diet, antibiotic therapy, prebiotic or probiotic supplementation, phage therapy and gut microbiota transplantation.

Studies have found that high-salt diet can enhance the function of natural killer (NK) cells by enriching the abundance of *Bifidobacterium*, thus inhibiting tumor growth (63). High dietary fiber can enrich *A. muciniphila*, activate innate immunity, reshape the tumor microenvironment, and exert the function of inhibiting tumor (64). Notably, *Wargo* et al. have confirmed that high-dietary fiber diet can enhance anti-tumor immunity and increase the infiltration of tumor-killing T cells, while commercial probiotics treatment alone does not enhance the efficacy of immunotherapy. This study suggests that probiotics intervention is strain-specific and should be put in a specific dietary environment to make sense, to some extent (65).

Oral administration of different antibiotics can significantly affect the corresponding gut microbiota. The duration of antibiotics use will affect the composition of gut microbiota, which have a profound impact on the occurrence and prognosis of tumors. It is found that oral antibiotics can significantly inhibit the growth of melanoma, pancreatic cancer and other tumors, which is related to the depletion of gut microbiota that increases the percentage of T cells in the tumor microenvironment and decreases the pro-inflammatory cytokines such as IL-17A and IL-10. It suggests that modulating gut microbiota can be a new combined strategy of tumor therapy (66). Other studies have shown that exposure to broad-spectrum antibiotics can lead to dysregulation of gut microbiota of mice, increase the relative abundance of *Proteobacteria* and the intestinal permeability, activate the NF- κB-IL6-STAT3 inflammatory pathway, and promote the growth of tumor and chemotherapy tolerance (67). However, removal of intestinal fungi by specific antifungal agents can enhance the efficacy of tumor radiation therapy, whereas removal of gut microbiota reduces the response of radiation therapy, which is associated with depletion of bacterial, leading to fungal overgrowth (68).

As the host commensal microbiota, the growth preference of gut microbiota has individual differences and high selectivity. Targeted supplementation of prebiotics and probiotics can effectively improve the colonization and growth of bacterial strains, to achieve the intervention of the structure of gut microbiota. Recent studies have revealed that Camu-Camu, a berry rich in polyphenols, contains ellagitannin, which can enrich the abundance of *Ruminococcaceae* and *Alistipes*, increases the infiltration of CD8^+^T cells in the tumor microenvironment, and enhances the efficacy of tumor immunotherapy (69). Similarly, pectin is a prebiotic that promotes the growth of butyrate-producing bacteria, thereby enhancing the efficacy of immunotherapy through increasing butyrate levels to promote the function of CD8^+^T cells (70).

Probiotics supplementation can inhibit tumor progression and enhance the efficacy of immunotherapy. Supplementation with *Bifidobacterium* or *Lactobacillus eosinophils* can modulate the tumor microenvironment and increase the infiltration of effector T cells. Studies have found that specific metabolites produced by some probiotics inhibit tumor growth. For example, *Lactobacillus delbrueckii subsp. bulgaricus* can produce EPS-R1, an extracellular polysaccharide that can induce CCR6^+^CD8^+^T cells, thereby enhancing the efficacy of ICI (71). Reuterin, the metabolite of *Lactobacillus reuteri*, inhibits CRC by altering the redox balance of tumor cells (72).

Surprisingly, fecal microbiota transplantation has been conducting out in preclinical and clinical studies with promising results. By means of germ-free and humanized tumor-bearing mouse models with gut microbiota, research demonstrate that specific gut microbiota plays an important role in tumor therapy. A clinical study of an Israeli team reveals that gut microbiota from responders can improve the efficacy to anti-PD-1-based immunotherapy for non-responders (73). This clinical research reports the similar finding, in which a single dose of fecal microbiota transplantation (FMT) combined with anti-PD-1 drug therapy is able to alter the gut microbiota of nonresponsive patients and assist patients in overcoming immunotherapy resistance by enhancing peripheral and intratumoral immune responses (73). There is no doubt about the efficacy of FMT therapy. However, there are still some problems remain to be solved in improving the effectiveness of gut-microbiota modulation. Firstly, it is challenging to isolate the effective bacteria from the donor and identify potential patients who required FMT therapy. Secondly, it is also important to evaluate the compatibility of the transplanted microbiota between the donor and the recipient, as the validation of FMT is associated with the efficiency of colonization. In addition, it is significant to determine the time-point of intervention and course of FMT, for better clinical therapy. The screening for “super donor” for patients with cancer would be a vital step to develop FMT as a clinical therapy, and how to combine dietary intervention or prebiotics with FMT also needs to be illustrated in further research.

## 5 The mechanisms of gut microbiota in tumorigenesis and progression

### 5.1 The stimulator of interferon genes (STING) signaling

Pattern recognition receptors (PRRs) are one of the main mediators between gut microbiota and host immune system. In recognition of microbial antigens, PRRs activate the intestinal immune system through a downstream cascade of signaling molecules. Several of these PRRs have been implicated in colitis-associated carcinogenesis, including Toll-like receptors (TLRs), nucleotide binding oligomeric receptors, Retinoic acid-inducible gene I (RIG-I) like receptors, and the missing melanoma 2-like receptor. Specifically, *F. nucleatum* can activate TLR4 signaling to promote tumor development, and another over-presented bacterium, *Peptostreptococcus anaerobius*, can promote carcinogenesis by activating TLR2 and/or the TLR4 pathway in patients with CRC.

Gut microbiota, as the commensal microbiota of the host, is closely related to the immune environment homeostasis. As the extrinsic microorganism, microbiota contains a variety of substances, such as DNA, RNA and protein, which act as antigens to activate the innate and adaptive immunity of the host; therefore, it plays a crucial role in tumor immunity (74). The innate immune system is the frontline of defense for the host’s immune system against foreign pathogens, which is mainly composed of NK cells mononuclear macrophages cells, DCs, granulocytes (75). As the effective cytokine produced by innate immune cells, type I interferon (IFN-Ι) is known to play an important role in anti-tumor effect and the defense against infection by pathogenic microbial. Recent studies have found that gut microbiota can promote the secretion of type I interferon through the activation of cyclic GMP-AMP (cGAS)-STING-IFN-I signaling pathway, thereby enhancing the anti-tumor effect. Supplementation with *Bifidobacterium* can activate STING signal through its accumulation in local tumor microenvironment, thereby promoting antigen presentation of DC cells and enhancing the anti-tumor effect of CD47 inhibitor (76).

The composition of tumor microenvironment often affects the efficacy of immunotherapy. Studies have shown that the tumor microenvironment (TME) in mice lacking microbiota shows an increase in tumor-promoting macrophages, and a decrease in monocytes and dendritic cells with anti-tumor effects (64). It suggests that microbiota can reshape the TME and affect the differentiation of mononuclear macrophages. Specific bacterial flora can produce cyclic adenosine diphosphate, which can bind with STING, activate the signaling pathway, and then promote the production of IFN-I by intratumor monocytes, thereby regulating the polarization of macrophages, promoting the interaction between NK cells and DCs, and further enhancing anti-tumor effects. Since high-fiber diet is known to over-represent the probiotics, a high-fiber diet can significantly increase the abundance of *A. muciniphila*, which can produce C-di-AMP and enhance the efficacy of ICIs (64).

A study has found some probiotics, including *Lactobacillus plantarum* and *Lactobacillus acidilacticii*, can activate inflammatory NF-κB signaling pathways and IFN-Ι response from macrophages, which is mediated by cytoplasmic receptor antiviral STING and mitochondria signal perception. Activation of downstream signaling molecules induces the expression of IFN-Ι (77). In addition, studies have found that *Lactobacillus rhamnosus* can produce IFN-Ι by promoting STING signaling pathway in DC cells, enhancing the activity of tumor killing CD8^+^T cells, and further promote the efficacy of immunotherapy (78). As commensal bacteria, some pathogens often play dual roles in the occurrence and progression of tumors. Studies have found that probiotics can activate the STING pathway and play a positive role in the immunotherapy. *F. nucleatum* activates STING signaling pathway, then induces the expression of PD-L1 in CRC cells, increases the infiltration of IFN-γ^+^CD8^+^T cells, and improves the sensitivity of tumors to PD-L1 immunotherapy (49).

### 5.2 Metabolites

As the commensal residents in the gut, gut microbiota can interplay with the host, and contributes to the metabolism of the host. It is known that gut microbiota transforms different nutrients into small substances and obtains what they require for survival and growth. However, these microbial metabolites as well as the associated products that bacteria mediated, exert great influence on the physical health (**Table 2**). Studies report that substances including microbes-associated small molecules and metabolites promote the tumorigenesis of the host and influence the response of treatment, through local and systemic effects (86). Metabolism function of microbiota is the vital mediator to interact the host and the microbes, which can play a profound role in the physiological function and immune environment (87). The metabolites of gut microbes derive, as well as the physical metabolism that microbes take part in, mediate the initiation and progression of different types of cancer, and the related treatments through different mechanisms.

**Table 2:** The metabolites of gut microbiota in cancer.

Study	Metabolites	Organism	Effect	Pathway
Luu, M.H. et al He, Y. et al	Short-Chain Fatty Acids (SCFAs), e.g., butyrate or propionate, etc	Some *Clostridium* genera or *Akkermansia muciniphila*	Anti-cancer	Mammalian target of rapamycin (mTOR) activity and autophagy by inhibiting histone deacetylases 3 (HDAC3) activity (79, 80)
Ridlon, J. M. et al	Bile acid, e.g., Taurocholic acid	*Clostridium seagrass*	Pro-cancer/Anti-cancer	Epidermal growth factor receptor (EGFR)-MAPK signaling; p53 level; β-catenin activation (81)
Yoshimoto, S. et al	Bile acid, e.g., Deoxycholic acid (DCA)	*Clostridium cluster* XI and XIVa	Pro-cancer	Senescence-associated secretory phenotype (SASP) (82)
Lin, R. et al.	Bile acid, e.g., Deoxycholic acid (DCA)		Anti-cancer	Expression of phosphatase and tensin homolog (PTEN) (83)
Wang, H. et al.	Trimetlylamine N-oxide (TMAO)	*Clostridiales*	Anti-cancer	Endoplasmic stress kinase PERK (84)
Mager, L. F. et al.	Inosine	*Bifidobacterium pseudolongum*	Anti-cancer	Th1 activation and antitumor immunity (85)

#### 5.2.1 Short-chain fatty acids (SCFAs)

SCFAs, the primary products of dietary fiber by gut bacterial fermentation, including acetate, butyrate, propionate, have been proved to exert profounding effects on the homeostasis of intestinal barrier and related immune function (88). Derived by some *Clostridium* genera or *A. muciniphila*, SCFAs can stimulate the generation of anti-inflammatory regulatory T-cell (Treg) cells to maintain the balanced immune environment (89). For example, the histone deacetylases (HDACs) inhibitory activity of butyrate, resulted in decreased expression of proinflammatory cytokines. It affects mammalian target of rapamycin (mTOR) activity and autophagy by inhibiting HDAC3 activity. Meanwhile, the inhibition in HDAC3 of CD8^+^T cells by butyrate and propionate, promotes the gene expression of effector molecules, such as IFN-γ and granzyme B, which can enhance the anti-tumor effect of the host, and improves the response to chemotherapy (79, 80). Similarly, SCFAs can also enhance the efficacy of cancer immunotherapy by enhancing the effector molecules through metabolic and epigenetic reprograming (90). Clinical research also verifies the positive role of SCFAs that patients with better response to ICI treatment have higher concentration of SCFAs *in vivo*, compared with those with poor response (91).

However, it remains controversial about the role of different types of SCFAs in the occurrence and development of cancer. There is a study demonstrating that acetate contributes to the tumor growth, through its biosynthesis of acetyl-CoA, which provides energy for cancer cells under hypoxia (92). It suggests that the detailed mechanisms about the function of SCFAs need to be further studied and its specific effects might depend on the specific context of tumor microenvironment (89). It is reported that pectin supplement significantly enhances the anti-PD-1 efficacy in tumor-bearing mice humanized with gut microbiota from patients with colorectal cancer (70). Given the production of SCFAs derives from the dietary fiber metabolism by specific bacteria, the strategy of supplementation treatment should be considered as the combination of probiotics and prebiotics, to achieve the feasible treatment effects for cancer therapy.

#### 5.2.2 Bile acid

In addition to dietary nutrients, commensal bacteria of the gut can modify and transform host-derived molecules into biologically active substances, such as the bile acid. As is known, bile acid is synthesized by the liver, stored in the gallbladder, and processed by gut microbiota, such as *Clostridium seagrass*, to produce deoxycholic acid (DCA) (93). Studies have demonstrated that high-fat diet can promote the synthesis of bile acid in liver. Excess secreted bile acids may enter the colon and promote the conversion of primary bile acids to secondary bile acids by 7α-dehydroxylation of colonic bacteria, to generate high levels of tumor-promoting DCA. It is reported that *Clostridium*, with the activity of 7α-dehydroxylase, can produce secondary bile acid DCA. DCA plays an important role in different tumors by regulating microRNA, enhancing EGFR-MAPK signaling pathway, and reducing p53 level and increasing β-catenin activation (81). For example, it is reported that supplementation with DCA led to an increased occurrence of hepatocellular carcinoma in a high-fat diet, while using antibiotics can reverse it (82).

However, there are studies demonstrating the anti-tumor role of DCA in different types of cancer. Research show that reduced DCA level is associated with poorer survival outcome in patients with gallbladder cancer (GBC) (83). Surprisingly, DCA inhibits tumor growth by inhibiting cell proliferation. In terms of mechanism, DCA reduces the expression of Mir-92b-3p by promoting the dissociation of methyltransferase-like 3 (METTL3) from the METTL3-MeTTL14-WTAP complex, thereby increasing the expression of phosphatase and tensin homolog (PTEN) tumor suppressor deleted on chromosome 10. DCA has been found to inhibit the growth of GBC by affecting the expression of PTEN. Therefore, DCA can be considered as a cancer suppressor in GBC. Although contradictory findings suggesting both protective and cytotoxic effects of DCA on cells, the current study demonstrates that the effects of DCA on cell fate are determined by cell types. Further research involved in interaction between microbiome and secondary BAs metabolome in cancer is valuable.

#### 5.2.3 Trimethylamine N-oxide (TMAO)

TMAO is a gut microbial-dependent metabolite from fat and dietary meat, which has been shown to be associated with higher risk of cardiovascular diseases (94). Importantly, the level of TMAO is associated with cancer risk, which may result from TMAO-induced inflammation. TMAO can activate reticulum stress kinase PERK-mediated unfolded protein responses, thereby inducing the transcription factor Forkhead box protein O (FoxO)1, a key regulator of metabolism (95). Thus, changes in the composition of gut microbiota and the level of TMAO are associated with the risk of cancer. However, a study demonstrates that patients with triple-negative breast cancer who have higher plasma TMAO achieve better responses to immunotherapy. Mechanistically, this metabolite can activate the endoplasmic stress kinase PERK, which enhances the function of CD8^+^T cells mediated immunity (84). Therefore, further understanding the role of TMAO in cancer formation will help determine how to prevent cancer through changes in diet, microbiota, and TMAO signaling in cancer control and prevention.

#### 5.2.4 Inosine

Research have demonstrated that three bacterial species, *Bifidobacterium pseudolongum*, *Lactobacillus johnsonii*, and *Olsenella*, influence the efficacy of ICIs (85). Remarkably, *B. pseudolongus* can enhance the efficacy of immunotherapy through producing the metabolite inosine, which is a metabolite that promotes immunotherapy. Mechanistically, inosine promotes Th1 activation and antitumor immunity which improves the anti-tumor effects. Therefore, inosine-based therapy may improve the efficacy of ICI. Collectively, the gut microbiota can influence the composition of metabolites in the gut, thereby contributing to genetic and epigenetic changes that lead to cancer. Thus, combining metabolite and microbiome analyses to elucidate the interactions between gut microbiota, metabolism, and host is essential for understanding microbiota how to regulate the tumorigenesis.

In conclusion, the gut microbiota is closely related to the physiological functions of the host. Microbiota often regulate the immune of the host through rewired metabolism, and then affect the process of tumor prevention and treatments. It is crucial to identify the effective bacteria and uncover the underlying mechanisms. However, even though current studies have confirmed that gut microbiota has beneficial effects in cancer prevention and treatment, the mechanism remains to be further studied.

## Author contributions

J-TH and Y-QM carried out the primary literature search, drafted and participated in discussions. Y-QM helped modify the manuscript and provided fund support. All authors contributed to the article and approved the submitted version.

## Funding

This work was supported by grants from National Natural Science Foundation of China (81872245 and 81803601), Research Project of Shanghai Municipal Health Commission (20214Y0328), Open Research Fund of State Key Laboratory of Genetic Engineering, Fudan University (SKLGE-2112).

## Conflict of interest

The authors declare that the research was conducted in the absence of any commercial or financial relationships that could be construed as a potential conflict of interest.

## Publisher’s note

All claims expressed in this article are solely those of the authors and do not necessarily represent those of their affiliated organizations, or those of the publisher, the editors and the reviewers. Any product that may be evaluated in this article, or claim that may be made by its manufacturer, is not guaranteed or endorsed by the publisher.
